# Sacrificing cells of the cyst: non-apoptotic cell death in germline cysts via acidification

**DOI:** 10.3389/fceld.2025.1677498

**Published:** 2025-10-28

**Authors:** Shruthi Bandyadka, Logan Tohline, Georgette-Vanelle Wandji, Hila Toledano, Kimberly McCall

**Affiliations:** 1 Department of Biology, Boston University, Boston, MA, United States; 2 Program in Bioinformatics, Boston University, Boston, MA, United States; 3 Medical School, University of Haifa, Haifa, Israel; 4 Department of Human Biology, Faculty of Natural Sciences, University of Haifa, Haifa, Israel

**Keywords:** germline cyst, ovary, testis, phagoptosis, phagocytosis, *Drosophila*

## Abstract

Cell death shapes multicellular organism development and sustains tissue and organ homeostasis. Great progress has been made in understanding the breadth of physiological and biochemical diversity in cell death and clearance pathways, which play vital roles in organismal development and health. While apoptosis and necrosis have been studied extensively across many model systems and contexts, the discovery of non-apoptotic paradigms of cell death and their roles in disease has greatly expanded the field. Collectively called Regulated Cell Death, these death pathways operate in a tissue and context-dependent manner. Germ cells in many organisms develop in cysts of interconnected cells, and may die in response to environmental or developmental cues. Recent findings suggest that germ cell cysts may use a common mechanism of non-apoptotic cell death involving phagocytic and lysosomal activity of surrounding somatic cells. Cyst cell death has been analyzed most thoroughly in the *Drosophila* adult ovary and testis, with remarkable similarity to cyst cell death in mouse adult testis and fetal ovary. In this review, we highlight recent progress in deciphering germline cyst cell death.

## Plethora of cell death pathways

For a multicellular life to sustain itself, parts of it must die many deaths in its lifetime. From eliminating excess cells in development, to promoting cell turnover to prevent disease, cell death has emerged as a sentinel and sculptor in shaping the destinies of individual cells within multi-cellular organisms (Fuchs and Steller, 2011; Ghose and Shaham, 2020). Armed with only a light-microscope, scores of scientists have witnessed and described dying cells or “necrobiosis” for at least a century (Lockshin, 2023). In 1972, Kerr, Wyllie, and Currie painted one of the most definitive pictures of death – pyknotic nuclei, indicative of chromatin condensation, and membrane-bound bodies shed from the dying cell, now known as “blebs” (Kerr et al., 1972). These tell-tale signs are now better understood as apoptosis, a form of cell death mediated by the serial cleavage and subsequent activation of caspase proteases. Another defining characteristic of apoptosis is the loss of lipid asymmetry on the dying cell’s surface and the abundant exposure of phosphatidylserine (PtdSer) on the outer leaflet of the plasma membrane. Apoptotic cell death therefore can be detected by staining for cleaved caspase-3 or the PtdSer-binding protein, Annexin V (Vermes et al., 1995; Julien and Wells, 2017). Research in the past decade has greatly expanded our knowledge of apoptosis, and has further identified alternative means of cell removal. Examples of non-apoptotic death include necrosis, which is characterized by organelle swelling and membrane rupture, and ferroptosis, in which excess iron accumulation and lipid peroxidation causes cell mortality without exhibiting the morphological markers associated with apoptosis or necrosis (Galluzzi et al., 2018; Li et al., 2020). Grouped under the umbrella term Regulated Cell Death (RCD), several non-apoptotic forms of cell death have been described, necessitating improvements to the taxonomy and methods for the detection of distinct pathways (Kroemer et al., 2009; Galluzzi et al., 2018; Tang et al., 2019). How specific non-apoptotic death pathways diverge from apoptosis, especially in the same multicellular system, is of particular interest.

### Cell clearance

Nearly all forms of RCD result in cell corpses and debris, which may cause inflammation if left uncleared (Rock and Kono, 2008). Molecules exposed on the dying cell’s surface, such as PtdSer and calreticulin, and those released by these cells such as nucleotides and S1P, referred to as “eat-me” and “find-me” signals respectively, attract phagocytes - cells that are capable of engulfing and digesting dying cells and other undesirable entities like pathogens (Arandjelovic and Ravichandran, 2015; Mehrotra and Ravichandran, 2022). Depending on their functional specificity and jurisdiction within the body, three classes of phagocytes have been recognized (Arandjelovic and Ravichandran, 2015; Freeman and Grinstein, 2016). “Professional phagocytes” are defined as cells whose primary function is to perform surveillance and clearance. Examples include macrophages, neutrophils, and dendritic cells, comprising core components of the innate immune system. These cells are recruited to sites of infection or injury to clear pathogens or damaged tissues, respectively. If infection and inflammation persist, these cells can escalate their response through activation of the adaptive immune response.

In contrast, “non-professional phagocytes” are employed typically during cell turnover events under homeostatic conditions, which may not require a major immune response. These include epithelial cells, that typically provide structural integrity to tissues and have been shown to perform cell clearance when necessary. Seeberg et al. (2019) further note that phagocytosis could be a general capability that could be triggered in a wide range of cells previously thought to be non-phagocytic. Finally, in the tissues that are inaccessible to professional phagocytes, there are highly specialized resident cells that have evolved to perform analogous roles. For example, the routine turnover of the photoreceptor outer segment is facilitated by clearance performed by the retinal pigment epithelium (Arandjelovic and Ravichandran, 2015). The general mechanism of engulfment and corpse processing within phagocytes is thought to be comparable across the different classes of phagocytes. Whether they interact during clearance and how their interaction may be orchestrated remains poorly understood.

### Phagoptosis

Phagocytes have been known to engulf their pathogenic “non-self” targets alive, while aiding in the removal of their kin destined for death. Defying convention that “self” cells are only cleared when they are dead or dying, Brown and Neher (2012) showed that viable cells may also be engulfed, which would otherwise continue to live, if not for being eaten alive. This form of cell death, where the phagocytosis apparatus of one cell is harnessed to kill another cell has been coined phagoptosis (Brown and Neher, 2012; 2014). In phagoptosis, the phagocyte kills the target cell, whereas in phagocytosis the phagocyte clears away the dead or dying cell. Despite the infancy of phagoptosis research, it has been investigated in multiple tissue systems (Lebo and McCall, 2021; Brown, 2023) and *Drosophila melanogaster* has emerged as a strong model organism to contextualize and characterize the phenomenon.

## Germ cell cysts

**FIGURE 1:**
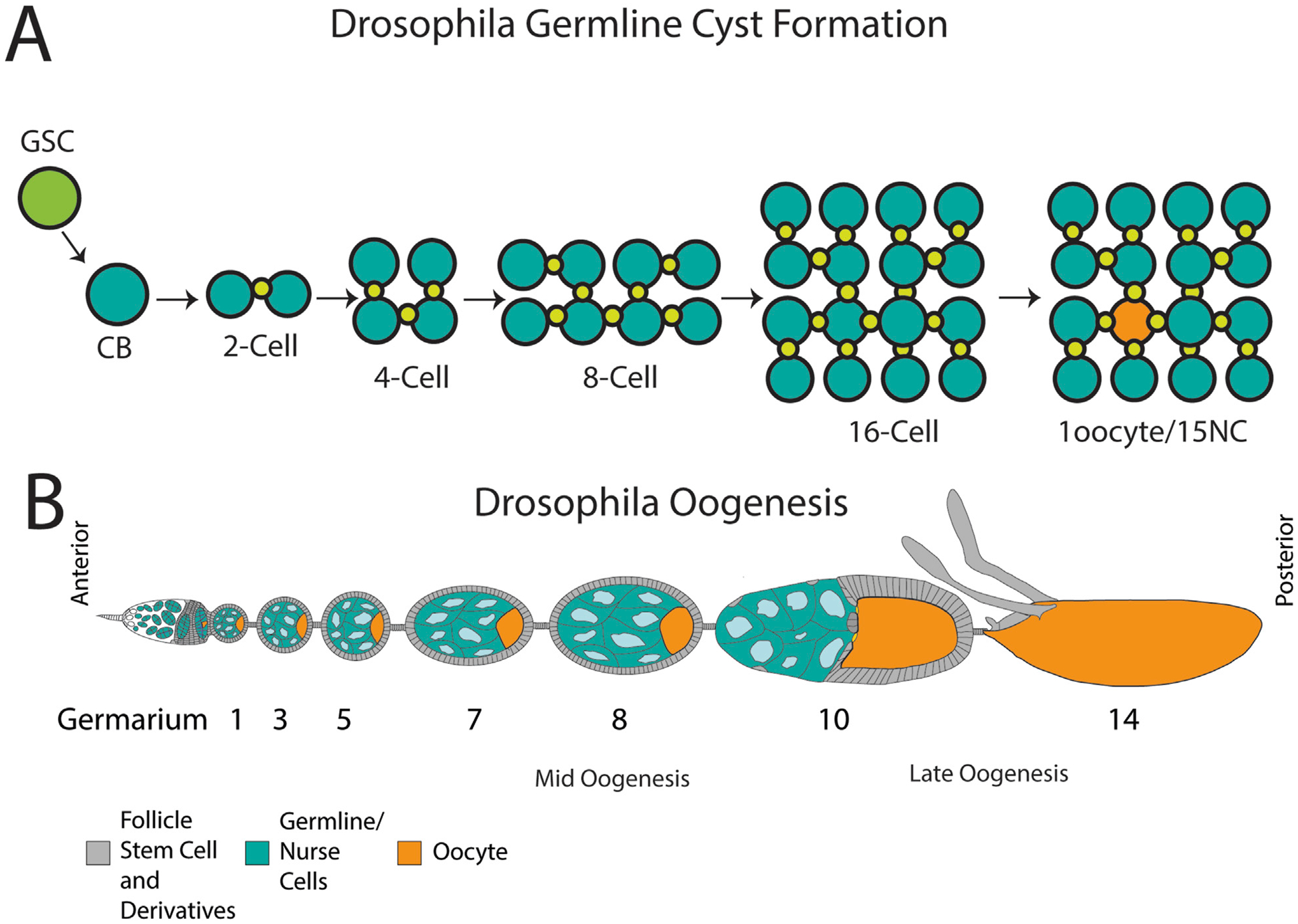
Cyst formation and organization of the *Drosophila* ovary. **(A)** A germline stem cell (GSC) divides unequally to form a cystoblast (CB). The CB divides into a two-cell cyst connected by an intercellular bridge or ring canal (green). The cysts continue their incomplete divisions for a final formation of a 16-cell cyst that will differentiate into an oocyte (orange) and 15 nurse cells (teal). **(B)** An ovariole with developing egg chambers, commencing with the germarium which houses stem cells and early cysts. The egg chambers progress through 14 stages of development, containing nurse cells (blue) and an oocyte (teal) surrounded by follicle cells (grey). Stages where germ cell death occur are indicated: mid-oogenesis and late oogenesis. By stage 14, the NCs have been cleared and a mature oocyte is formed. Schematic in B modified from Lebo and McCall (2021).

One surprising tissue where phagoptosis has emerged as a major cell death mechanism is the germline of multiple organisms. In both the ovary and testis of many organisms, pre-meiotic germline cells form in cysts. Germline stem cells give rise to daughter cells that divide via incomplete cytokinesis, forming cysts of cells connected by intercellular bridges (Figure 1A). These cysts are surrounded by somatic cells that are generally thought to protect the cyst and promote the development of the germ cells. Germline cysts are found in male germlines of most if not all animals, suggesting an ancient origin (Spradling, 2024). In females, germline cysts are found in diverse animals including mammals and insects. In the testis, all cells of the cyst differentiate into spermatocytes, whereas in the ovary, typically one cell in the cyst is specified as the oocyte, with other cells developing as nurse cells that support the oocyte (Spradling, 2024). However, there is substantial diversity in ovarian germline structure among animals (Brubacher, 2024). Cell death is used as a “quality control” mechanism in the germline, but the interconnected cyst presents a challenge to eliminate without causing damage to the tissue. Recent findings point to similar cell death mechanisms being used to eliminate germline cyst cells in both the ovary and testis of diverse organisms.

## Apoptotic and non-apoptotic death in *Drosophila* oogenesis

*Drosophila* oogenesis is highly regulated and requires the precise spatiotemporal coordination of germline and somatic cells. Under optimal conditions, each viable oocyte progresses through 14 stages of development within the ovary (Figure 1B). The germline-derived oocyte and nurse cells (NCs) are surrounded by a monolayer of somatic follicle cells (FCs) to form an egg chamber; each population has distinct roles, working together to monitor and promote egg development (King, 1970; Spradling, 1993).

Germline cell death can be triggered at two distinct stages of development after the germarium stage, with each paradigm indicating a distinct fate for the oocyte (Lebo and McCall, 2021; Bandyadka et al., 2025). The first of these forms of cell death occurs in mid-oogenesis (Figure 1B). Under unfavorable or stressed conditions, cell death can be induced by a key checkpoint roughly half-way through oogenesis between stages 7–9, causing the entire egg chamber to degenerate (Giorgi and Deri, 1976; Buszczak and Cooley, 2000; Drummond-Barbosa and Spradling, 2001; Serizier and McCall, 2017). During this midstage death, FCs engulf the oocyte and NCs (Giorgi and Deri, 1976; Etchegaray et al., 2012), which undergo a form of apoptotic and autophagic death (Laundrie et al., 2003; Mazzalupo and Cooley, 2006; Hou et al., 2008; Nezis et al., 2009). Draper (Drpr), a phagocytic receptor homologous to mammalian MEGF10 and *C. elegans* Ced-1, is expressed on FC membranes during midstage death and recognizes the apoptotic germ cells, activating JNK signaling to promote cell clearance (Etchegaray et al., 2012).

**FIGURE 2:**
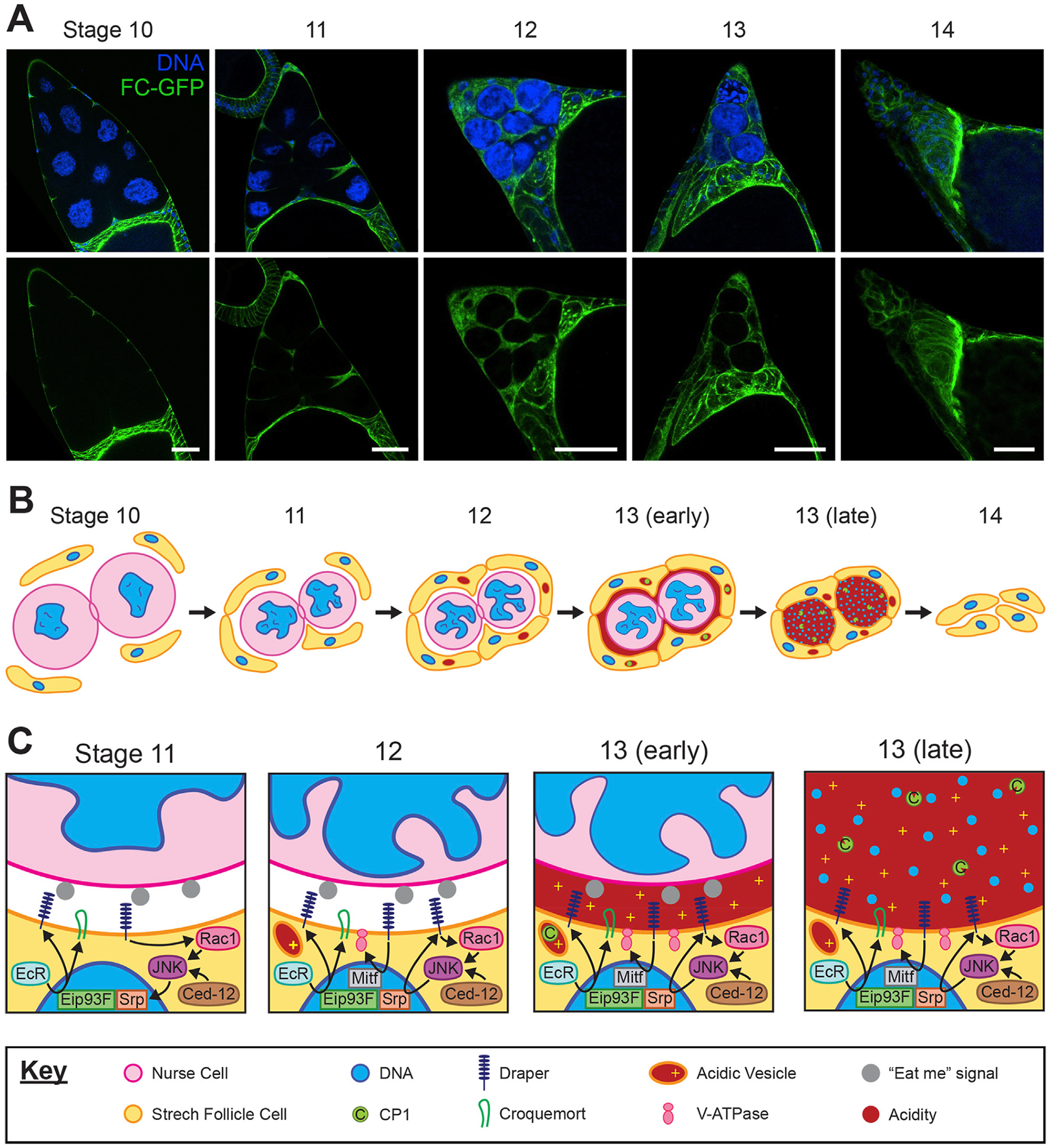
Development and interaction of stretch follicle cells with nurse cells during late oogenesis. **(A)** Representative confocal images of the anterior end of *Drosophila* egg chambers from stages 10–14. Asynchronous clearance of nurse cells (larger nuclei) is completed by stage 14 of normal development. Follicle cell membranes are labeled by expression of *traffic jam-Gal4; UAS-myrGFP* (green) and nuclei are labeled with DAPI (blue). Scale bars = 50 μm. **(B)** Progression of stretch follicle cell clearance of nurse cells from stages 10–14. A ring canal is depicted connecting the two nurse cells. **(C)** Closeup view of the junction between a follicle cell and nurse cell from stages 11–13, including follicle cell pathways which mediate nurse cell clearance. The key applies to panels **(B,C)**.

The second form of germ cell death in the ovary occurs in late oogenesis from stage 10–14 (Figure 1B) when the NCs are specifically eliminated. During stage 11, the supportive nurse cells “dump” their cytoplasmic contents into the oocyte via their ring canals, including organelles, proteins, and maternal mRNAs. NCs are left with nuclei and some cytoplasm (Spradling, 1993; Berg et al., 2024). The remaining nurse cell components are disposed of, with NC death progressing asynchronously from stages 12–14. This developmental NC death is non-apoptotic and non-autophagic; NC death occurs despite disruption of caspases and autophagy-related genes in the germline (Mazzalupo and Cooley, 2006; Barth et al., 2011; Peterson and McCall, 2013). By the end of oocyte development at stage 14, all NCs have been removed (Figure 2A) (Timmons et al., 2016).

During the latter stages of oogenesis prior to NC death, the egg chamber morphology changes as the oocyte grows and by stage 10 most FCs migrate to surround the oocyte, now termed main body FCs. A few FCs in the anterior region elongate and flatten, defined as Stretch FCs (SFCs), and these cells migrate and extend into the spaces between the NCs (Figure 2A) (Osterfield et al., 2017; Berg et al., 2024). These SFCs are key players in the death and removal of NCs at the end of oogenesis (Figure 2B).

Genetic ablation of the SFCs revealed that they are required for NC dumping and DNA fragmentation of the NCs, indicating non-cell-autonomous control over NC death (Timmons et al., 2016). Moreover, the nuclear lamina involutions that normally occur during NC death are inhibited following SFC ablation (Yalonetskaya et al., 2018). Strikingly, components of the phagocytosis machinery *drpr*, *Ced-12*, and the JNK signaling pathway are required within SFCs for developmental NC death, with mutants or knockdowns of these genes showing inhibition of the DNA fragmentation and nuclear lamina changes that occur during NC death (Timmons et al., 2016; Yalonetskaya et al., 2018). This requirement for phagocytosis genes classifies NC death as phagoptosis.

Many of the same follicle cells act as non-professional phagocytes during both stress-induced midstage death and healthy developmental NC death, with overlapping use of pathways such as those including Drpr, Ced-12 and JNK (Etchegaray et al., 2012; Timmons et al., 2016). Yet, the distinction of late-stage developmental NC death as non-cell-autonomously controlled and non-apoptotic highlights this as a unique instance of differential cell fates wherein the *Drosophila* ovary is an important model system.

### Stretch follicle cell transformation into phagocytes

The *Drosophila* ovary is considered immune-privileged, meaning hemocytes (macrophages) do not enter the ovary to act as professional phagocytes for clearance of dying NCs (Heron et al., 2023; Chasse et al., 2024). In late-oogenesis, NC remnants are removed efficiently by SFCs which take on the role of non-professional phagocytes and directly mediate the clearance of NCs; thus, proper SFC differentiation is required for effective oogenesis.

Prior to SFC differentiation, FCs switch their cell cycle to endocycling during stage 6 of development (Deng et al., 2001). This transition is marked by activation of Notch signaling across all FCs, and is important to differentiation of FC subtypes (Deng et al., 2001; Sun and Deng, 2007). Notably, Notch activation is premature in mutant clones lacking autophagy genes *Atg1* or *Atg13*, and oocytes do not complete proper development (Barth et al., 2012). Furthermore, polyploidy induced by Notch signaling is necessary for FCs to engulf in mid-oogenesis and for SFCs to effectively clear NC remnants (Huang et al., 2025).

Recently, a role for the hormone ecdysone was discovered in regulating phagocytosis during developmental NC death. Ecdysone signaling has been established as part of the initial transition of FCs into their fate as SFCs around stage 9, yet signaling remains active through stage 13. At stage 11, ecdysone bound to its receptor (EcR) induces the transcription factor (TF) Eip93F to localize into the nucleus, promoting transcription of *croquemort* (*crq*) and *drpr* (Figure 2C) (Ghosh et al., 2025). Draper enrichment on SFC membranes first becomes visible at stage 11, suggesting ecdysone triggers the initiation of expression of this phagocytic receptor. In stages 12–13, Drpr expression is increased and perpetuated by JNK signaling through a positive cycle, as well as through JNK activation by Ced-12 (Timmons et al., 2016). Additionally, ecdysone mediates the epithelial to mesenchymal transition (EMT) of SFCs, marked by the TF Serpent (Srp) (Ghosh et al., 2025). Srp was recently shown to be important for the expression of *drpr* in stages 12–13, acting downstream of JNK signaling to induce transcription of *drpr* (Zeng et al., 2025). In hemocytes, Srp is a key regulator of phagocytic capacity and activity, including for the expression of *crq* and *drpr* (Shlyakhover et al., 2018). The expression of Srp in SFCs may be an indicator of their transition to a phagocytic fate.

The role of Crq in oogenesis has not yet been thoroughly investigated. Despite an original characterization of Crq as a phagocytic receptor, a recent study showed that Crq is not required for engulfment during apoptosis. Crq is involved in phagosome maturation and degradation via lysosomes, possibly due to a role in lipid uptake (Woodcock et al., 2015; Westlake et al., 2025). Crq is required for proper clearance of NCs, as its downregulation led to some persisting NC nuclei (PNCN) at stage 14 (Ghosh et al., 2025). In lipid metabolism, JNK signaling functions downstream of Crq; it is possible the pathways connect similarly in oogenesis (Woodcock et al., 2015).

Another protein likely involved in lipid trafficking during SFC-mediated death is Eato, which affects both accumulation of Drpr along the SFC membrane and membrane stretching around NCs (Santoso et al., 2018). Eato is an ortholog of the ABC transporter Ced-7 in *C. elegans*, which contributes to lipid trafficking during engulfment of dying cells (Wu and Horvitz, 1998; Yu et al., 2006). In phagocytosis of *Drosophila* neurons, Eato influences the presentation of “eat-me” signals and phagocytes’ sensitivity to these signals (Chen et al., 2025). Although the exact role of Eato in the ovary is not known, it is likely part of the Drpr pathway, and functions in parallel to the Ced-12 pathway (Santoso et al., 2018).

While Drpr has been extensively characterized as a phagocytic receptor in both apoptotic and non-apoptotic death, the existence of an additional receptor has been hypothesized to be involved in phagoptosis (McPhee et al., 2010; Timmons et al., 2016; 2017). Transmembrane receptor integrins αPS3 and βPS are enriched on SFC membranes in stages 11–13, and may act upstream of Ced-12 (Timmons et al., 2017). Double mutants for both *drpr* and *αPS3* showed a higher number of PNCN than either mutant alone, indicating they function in parallel pathways (Timmons et al., 2017). Integrins are involved in the phagocytosis of apoptotic cells and bacteria in *Drosophila*, functioning to transmit signals (Nonaka et al., 2013; Sayedyahossein and Dagnino, 2013). It is possible integrins recognize a signal presented on NC membranes during developmental NC death, though this may or may not be the same signal recognized by Drpr. Other phagocytic receptors and proteins involved in detection of phagocytic targets in macrophages were screened via RNAi knockdown in SFCs, revealing high PNCN in NimC1, Eater, Tep4, and PGRP-LC lines (Ghosh et al., 2025).

Unlike many other forms of RCD where a single phagocytic cup is formed to endocytose the dying cell, developmental NC death involves the collaboration of four to five SFCs surrounding each NC (Figure 2B). Complete coverage by these SFCs is required for NC clearance (Ghosh et al., 2025). Thus, the phagoptosis of NCs is atypical: it requires the phagocytic machinery but does not proceed by typical engulfment by single phagocytes. Further understanding in the cooperation of these phagocytic cells may have implications for targeted cancer treatment, where harnessing cellular machinery to clear tumor cells is highly desirable (Huang et al., 2025).

In the final stages of NC clearance, Vacuolar ATPases (V-ATPases) become enriched on the plasma membrane of SFCs, promoting acidification of the NC remnants (Figure 2C) (Mondragon et al., 2019). Chloride channels are localized similarly, likely balancing the ion gradient formed by V-ATPase activity (Mondragon et al., 2019). V-ATPase protein complexes are typically involved in acidification of lysosomes and are present in the lysosomal membrane, although certain specialized cell types do target V-ATPase to the plasma membrane including human osteoclasts, renal intercalated cells, and even some tumor cells (Brown et al., 2009; Stransky et al., 2016; Futai et al., 2019). Interestingly, lysosomes do develop in the SFCs adjacent to NCs by stage 12, and are not observed in other FC subtypes (Cummings and King, 1970; Timmons et al., 2016; Mondragon et al., 2019). However, the V-ATPase does not appear to be localized to the plasma membrane via lysosomes; it is unknown how V-ATPase is transported in SFCs. The expression of V-ATPase genes is controlled through the TF Mitf (Zhang et al., 2015), knockdown of which leads to PNCN (Mondragon, 2019). Drpr is required for acidification of NCs, suggesting it may influence V-ATPase or Mitf expression. Following acidification, cathepsins such as CP1 produced by SFCs are released into the extracellular environment, where they are activated by the low pH (Mondragon et al., 2019; Yalonetskaya et al., 2020). Aided by DNaseII, the NC lamins and chromatin are degraded (Bass et al., 2009). Thus, the final demise of the NCs is mediated by extracellular acidification and released enzymes rather than typical phagolysosomal acidification following engulfment.

The developmental cell death of nurse cells has been considered a unique process, however recent findings in other systems suggest that this novel form of cell death may be conserved. These other systems are described below.

## Apoptotic and non-apoptotic death in *Drosophila* spermatogenesis

**FIGURE 3:**
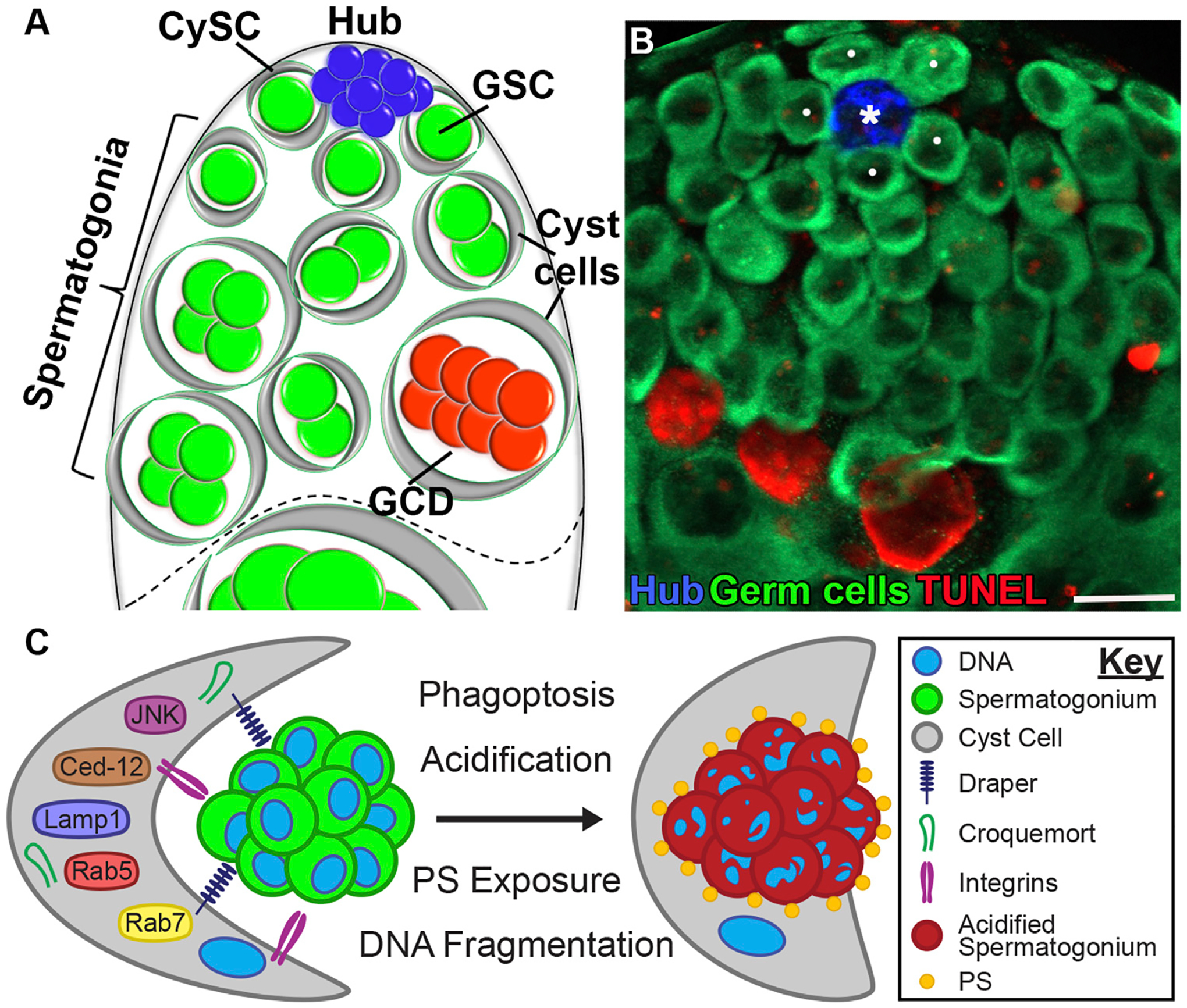
Spontaneous germ cell death (GCD) at the apical tip of the *Drosophila* testis. **(A)** Schematic representation of the apical tip of the testis (side view). The niche consists of GSCs (green) and CySCs (gray) both attached to the hub (blue). Spermatogonial germ cells (green), which are transit-amplifying progenitors, are encapsulated by early cyst cells (gray). Approximately one-quarter of spermatogonia undergo GCD (red). The dashed line demarcates the boundary between spermatogonia from terminally differentiated spermatocytes. **(B)** Wild-type testes (*w*^*1118*^) immunostained for TUNEL (fragmented DNA, red), Fas3 (hub, blue) and Vasa (germ cells, green). White dots mark GSCs; the asterisk denotes the hub. Scale bars, 10 μm. **(C)** Schematic model of germ cell phagoptosis. A cyst cell (gray) expresses phagocytic receptors (Draper and Integrins) that mediate the engulfment of a live spermatogonium (green). Following phagosome formation, PS is exposed, and the phagocytic machinery is recruited to degrade the engulfed contents.

The apical tip of the *Drosophila* testis houses two adult co-differentiating stem cell lineages–the germline stem cells (GSCs) which beget spermatogonial germ cells, and the somatic cyst stem cells (CySCs), which give rise to the epithelium that envelops the spermatogonia in cysts as they undergo transit-amplifying divisions. GSCs and CySCs are interspersed and directly contact a cluster of post-mitotic hub cells. The hub is a compact sphere of ~15 somatic cells that secrete short-range signals and express adhesion molecules to maintain the adjacent stem cells (Kiger et al., 2001; Tulina and Matunis, 2001; Leatherman and Dinardo, 2010). When a GSC or CySC divides, one daughter cell remains adherent to the hub to self-renew, while the other is displaced and undergoes transit-amplifying divisions before terminal differentiation into a spermatocyte or a differentiated cyst cell, respectively (Insco et al., 2009). Together, these 3 cell types, hub cells, CySCs, and GSCs, form the stem cell niche (Figure 3A).

Among the three, hub cells are the most resilient to cell death. Exposure of *Drosophila* to high doses of irradiation temporarily halts spermatogenesis and eliminates approximately half of the stem cells. Remarkably, however, the hub cells, despite being a central component of the niche, remain completely intact. This exceptional resistance to apoptosis is mediated by the expression of multiple anti-apoptotic microRNAs, including *bantam*, which are selectively expressed in the hub and render these cells inert to apoptotic stress signals (Volin et al., 2018). This pattern contrasts starkly with that of the *Drosophila* ovary, where *bantam* is expressed in the GSCs themselves rather than in the niche (Xing et al., 2015). This raises an intriguing question: why does protection from apoptosis reside in the hub cells of the testis, while in the ovary it is intrinsic to the GSCs? One possible explanation lies in the fundamental difference between the two systems regarding dedifferentiation. In females, GSCs are *bona* fi*de* stem cells established during embryogenesis, which continuously produce differentiated progeny throughout life. In contrast, in males, dedifferentiation of progenitor cells back into GSCs occurs regularly during adulthood (Brawley and Matunis, 2004). Thus, in the testis, protection from apoptosis is conferred by the hub cells, which not only serve as the structural core of the niche but also express adhesion molecules and secrete factors that promote dedifferentiation. This mechanism supports ongoing stem cell turnover and tissue regeneration in the male gonad.

In the testis, the term cyst has two distinct meanings. First, it refers to the somatic cyst cells that are generated following CySC division. Second, cyst is used to describe the differentiation unit, which includes the cyst cells that encapsulate and escort a cohort of clonally related spermatogonia. In this sense, male and female germline cyst units are conceptually similar, as they both refer to germ-cell collectives encased by supporting cells. Spermatogonial progenitors arise from four sequential divisions of GSCs, initially producing gonialblasts, the single daughter germ cells that are displaced from the niche following the asymmetric division of the GSCs. Each gonialblast undergoes mitotic transit-amplifying divisions with incomplete cytokinesis, forming spermatogonial clusters of 2, 4, 8, and 16 germ cells within individual cysts (Figures 3A,B) (Gönczy et al., 1997).

Just as protein deprivation triggers apoptosis of the germline in *Drosophila* mid-oogenesis, germline cysts undergoing transit-amplification in the testes have similarly been shown to undergo starvation-induced cell death, while preserving the competence of GSCs (Yang and Yamashita, 2015; Chiang et al., 2017). Under starvation, apoptosis is induced in the cyst cells, which in turn triggers the death of the encapsulated spermatogonia. The dying cyst cells are positive for cleaved caspase-3, confirming the activation of apoptotic pathways. In contrast, the spermatogonia that die as a result become positive for LysoTracker and TUNEL but remain negative for cleaved caspase-3, suggesting that their death does not occur via classical apoptosis. Instead, these findings point to a non-apoptotic mechanism of germ cell death initiated by the apoptosis of surrounding cyst cells.

Germline cell death during stress-free spermatogenesis in *Drosophila* bears a striking resemblance to NC phagoptosis during oogenesis (Yacobi-Sharon et al., 2013). Imaging of fixed testis samples suggests that 20%–30% of mitotic spermatogonial cysts undergo spontaneous germ cell death under homeostatic conditions (Figure 3B). Chiang et al. (Chiang et al., 2017) further estimated that this germ cell death process is prolonged, taking more than 24 h from initiation to completion. This extended duration may indicate that fewer germ cells are actively dying at any given time, but their persistence within the tissue makes them more readily detectable. Yacobi-Sharon et al. determined that loss of function of *Drice* and *Dcp-1* failed to reduce levels of cell death, thereby eliminating a role for effector caspase activation and establishing that developmental germ cell death in spermatogenesis is non-apoptotic. However, germ cell death requires an atypical function of Caspase-9/Dronc acting independently of its catalytic activity and its interaction with the apoptosome (Yacobi-Sharon et al., 2013; Napoletano et al., 2017). Autophagic death was also ruled out, as loss of function mutants of *Atg7* and *Atg8*, and overexpression of *Atg1* in spermatogonial cells did not elicit a strong cell death response. The first indication that the death of spermatogonial cysts involved acidification was due to the observation that dying cysts of mid- and late-spermatogenesis robustly co-stained with LysoTracker and TUNEL. A follow-up screen for mutants in lysosome-associated genes *CathD* (*cathepsin D*), *dor* (*deep-orange*), *car* (*carnation*), and *DNaseII* yielded a significant decrease in the number of dying cysts, squarely implicating lysosomes in the death of spermatogonial cysts (Yacobi-Sharon et al., 2013). In contrast to starvation conditions where apoptosis of cyst cells triggers germ cell death, inhibition of cyst cell apoptosis does not affect the spontaneously occurring germ cell death indicating that this mechanism is specific to starvation (Yang and Yamashita, 2015).

Following this discovery, a series of genetic screens and live imaging established that acidification was essential for germ cell death and that its control lay non-autonomously with the lysosomal secretions of the surrounding cyst cells (Figure 3C) (Zohar-Fux et al., 2022). The evidence for cyst cell involvement in germline acidification came from live imaging of Lamp1, a lysosomal membrane protein and an essential component of the phagocytosis machinery. Lamp1-GFP expressing phagosomes of cyst cell provenance were observed to aggregate in the interstitial regions between cyst cells and spermatogonial germ cells prior to LysoTracker staining, and disruption of Lamp1 in cyst cells with RNAi reduced the death of the germline - a necessary qualifier for phagoptotic death. Similarly, Rab5-YFP–and Rab7-YFP–positive endosomes in cyst cells were observed localizing around germ cells (Chiang et al., 2017), preceding LysoTracker and TUNEL signals (Zohar-Fux et al., 2022). These phagosomes surrounded the entire spermatogonium, indicating that the interconnected germ cells are engulfed as a single unit. Notably, RNAi-mediated knockdown of *Rab5* in cyst cells completely arrested germ cell death, further supporting the role of cyst cell–mediated phagoptosis in this process. Therefore, the post-mitotic cyst cells have two roles: (1) encapsulating germ cells throughout their differentiation and (2) clearing targeted germ cells through phagoptosis. It remains to be determined whether the cyst cell that encapsulates a specific spermatogonium is the one that induces its phagoptosis, or whether a remote cyst cell carries out the engulfment of the targeted spermatogonium.

Beyond acidification, spermatogonial death closely mirrored NC phagoptosis in morphology, as well as in the mechanism used for germline engulfment and clearance. Much like SFCs extending their processes around NCs, cyst cell membranes were observed to stretch into the regions where degrading germ cell chromatin was present post-acidification. The phagocytic receptors Drpr and Crq were also found to be highly expressed in cyst cells but were inferred to play complementary roles. Whereas *drpr* and *Ced-12* were determined essential for engulfment and did not impact acidification, *crq* RNAi only affected acidification without disrupting engulfment. Loss of *drpr* or *Ced-12* function leads to defective engulfment and results in hyperplasia at the apical tip of the testis, where unengulfed live spermatogonia accumulate, causing noticeable tissue enlargement. A similar phenotype was observed also in *p53* null mutants, suggesting a role for the tumor suppressor p53 in promoting germ cell death (Napoletano et al., 2017). In all cases, staining for phosphorylated histone H3 (pHH3) confirmed that the increased number of spermatogonia resulted from impaired clearance rather than excessive cell division. In the testes of *p53*^−*/*−^ mutant flies, the number of dying germ cells was reduced by approximately 33% and restored by overexpressing *p53* in spermatogonia on the null background, suggesting that *p53* can intrinsically induce germ cell death. In contrast, in mice, *p53* does not affect spontaneous germ cell death under normal conditions. However, under mild heat-shock, non-apoptotic germ cell death is significantly reduced in the testes of *p53*^−*/*−^ mice compared to wild-type, indicating an evolutionarily conserved role of *p53* (Napoletano et al., 2017). Notably, in contrast to the testis, loss of *drpr* or *Ced-12* in the ovary disrupts both acidification and engulfment in NC phagoptosis (Timmons et al., 2016; 2017; Yalonetskaya et al., 2018). Additional similarities to NC phagoptosis are that cyst cells activate JNK (Zohar-Fux et al., 2022) and require integrins for germ cell death (Perry et al., 2024), both reminiscent of SFCs in the ovary (Figure 3C) (Timmons et al., 2016; 2017).

In contrast to the multiple phagocytic receptors identified in the phagocytic cyst cells of the testis, no signal has yet been detected in germ cells that actively promotes their phagoptosis. In cells that are already dying or dead, target recognition typically occurs through exposed “eat-me” signals, with PtdSer being the most well-characterized. Under normal conditions, PtdSer resides in the inner leaflet of the plasma membrane, but it becomes externalized during apoptosis. Phagocytic receptors such as Drpr can bind directly to these signals or indirectly via bridging molecules. Following recognition, the process proceeds through phagosome formation, maturation, degradation, and eventual resolution of the engulfed cell (Melcarne et al., 2019). Although reversible exposure of PtdSer has been implicated in mediating phagoptosis in mammals (Brown and Neher, 2012), in the *Drosophila* testis, PtdSer is detected on germ cells only after they are engulfed by cyst cells (Zohar-Fux et al., 2022). This suggests that other “eat-me” signals may be expressed on live germ cells, mediating their recognition and phagoptosis independently of PtdSer exposure, though these signals remain to be identified.

In addition to spontaneous germ cell death, in which the entire spermatogonial cyst is engulfed as a single unit (Zohar-Fux et al., 2022), ionizing radiation also induces germ cell loss in the *Drosophila* testis. Under these conditions, the intercellular connectivity between spermatogonia within a cyst appears to function as a sensitizing mechanism, rendering the entire cyst susceptible to DNA damage.

Inhibiting cyst cell apoptosis did not suppress radiation-induced germ cell death, suggesting a germ cell intrinsic mechanism. However, this study preceded the identification of phagoptosis as the underlying mechanism of germ cell elimination, indicating that the observed radiation-induced death could have involved cyst cell mediated engulfment. In this context, blocking apoptosis might even alter the availability or behavior of cyst cells engaged in engulfment. Future experiments targeting the phagocytic machinery within cyst cells during irradiation could therefore be instrumental in clarifying whether irradiation-induced germ cell death truly reflects a cell-intrinsic response (Lu and Yamashita, 2017).

While the role of V-ATPases in NC phagoptosis is well-established, their involvement in spermatogonial death has not been investigated. It is likely that V-ATPases localize to cyst cell membranes to perform proton secretion akin to SFCs, and the mechanism by which they are trafficked to cyst cell membranes could also be conserved between the sexes. Nevertheless, the discovery of cyst cell phagoptosis of spermatogonial cells by acidification opens up exciting avenues for comparative studies on phagoptosis in *Drosophila*.

## Germline death in mouse oogenesis is non-apoptotic

An exciting recent discovery has shown that germline death and clearance in mouse oogenesis is a variation on the theme of phagoptosis by acidification. Like the *Drosophila* egg chamber, mouse germ cells develop in aggregates known as cysts. Primordial germ cells undergo mitotic divisions to form clusters of up to 32 synchronously developing, interconnected nurse cells (Pepling et al., 1999; Spradling et al., 2022). Mouse NCs perform functions analogous to *Drosophila* NCs. Maternal mitochondria are transferred to the oocyte from NCs in an aggregate known as the Balbiani body, following which NCs are eliminated in an all too familiar fashion.

Previously believed to be apoptosis (Morita and Tilly, 1999), Niu and Spradling (2022) showed that germline death in mouse ovaries is controlled by the phagocytosis machinery of the surrounding epithelial pregranulosa cells. LysoTracker activity was observed to increase in NCs around late-oogenesis. Further, inhibition of V-ATPase activity using Bafilomycin A1 in culture containing germline cysts, led to absence of LysoTracker activity and persistence of NC nuclei. Analysis of single-cell RNA-seq data showed late-oogenesis pregranulosa cells expressing several V-ATPase subunits and Cathepsins Ctsb, Ctsd, Ctsl (ortholog of *Drosophila* Cp1) and Ctsz. However, it is unknown whether this expression is temporally variable in oogenesis–starting with low expression in early-oogenesis pregranulosa cells and increasing after the transfer of NC cytoplasmic contents into the oocyte has taken place.

Niu and Spradling (2022) also make an important observation that apoptosis of germ cells also occurs simultaneously with phagoptosis, albeit at significantly lower levels and theorize that apoptotic death is used to remove non-viable germ cells. This observation is also consistent with studies of starvation-induced germline death in *Drosophila* gametogenesis. This suggests that there is preferential activation of apoptotic and non-apoptotic pathways, where specific cues and conditions lead to deterministic outcomes. Altogether, these exciting discoveries in *Drosophila* and mouse models indicate that phagoptosis by somatic support cells is a conserved mechanism for germline removal in gametogenesis.

## pHinal countdown: acidification as a cell killing mechanism could be unique to gametogenesis

These findings also raise important questions on the nature of phagoptosis, such as determining the necessary and sufficient conditions required to mediate this form of cellular assassination, the conditions under which it evolved, and the consequences when it goes awry. Phagoptosis was first characterized in neuronal death involving microglial phagocytosis (Brown and Neher, 2012; 2014). Stressed, but otherwise competent neurons exposing PtdSer on their membranes were phagocytosed by microglia. However, blocking microglial phagocytosis or reversing PtdSer exposure on the neurons prevented their engulfment, indicating that neuronal survival was controlled extrinsically by the phagocytosis machinery of microglia. Beyond PtdSer, other molecules such as calreticulin, which are typically considered as “eat-me” signals released by apoptotic cells, have also been identified on viable cell surfaces, leading to their phagocytosis by neutrophils (Gardai et al., 2005; Brown, 2023). Taken together, it is evident that there is diversity in the prerequisite conditions and the mechanisms whereby cell death via phagocytosis occurs.

Whereas cell death in the *Drosophila* testis appears to proceed by a “typical” phagoptosis mechanism where engulfment by a single cell occurs prior to phagolysosomal acidification, NC death in the ovary occurs by an alternative phagoptotic mechanism involving the phagocytic machinery of multiple cells promoting external acidification of a much larger target cell. Intriguingly, external acidification does not appear to play a death-associated role in other contexts. This suggests that phagoptosis by acidification is a special case of cell clearance, evolved specifically in gametogenesis. Extracellular proton secretion by plasma membrane expression of V-ATPases has been previously shown to occur in contexts outside of cell death. In rodent kidneys, epithelial intercalated cells have been shown to express V-ATPases on their plasma membranes to regulate pH homeostasis in the collecting tubule (Al-Awqati, 2013; Eaton et al., 2021). Both proton secretion by V-ATPases and cathepsin function is known to be essential for bone resorption and hydrolysis of the bone matrix (Toyomura et al., 2003). The exceptionally diverse functions and equally impressive structural conformations and localization patterns of V-ATPases have been comprehensively reviewed elsewhere (Futai et al., 2019; Eaton et al., 2021), but their involvement in cell killing thus far appears to be constrained to gametogenesis.

From an evolutionary perspective, this seems bewilderingly paradoxical at first glance. Why would cysts evolve an evidently destructive method to remove the germline, given its close proximity to the developing gamete? Given that reproductive fitness is the very foundation of evolution, a precariously incubated gamete seems counter-intuitive. One possibility is that while acidification may be detrimental to NCs, it may not impair the gamete, but may even be beneficial for its development and competency. The male reproductive tract in rodents maintains an acidic environment to aid nascent sperm attain fertility and maturation, unsurprisingly by V-ATPases performing extracellular proton secretion (Breton et al., 1996; Shum et al., 2009; Dai et al., 2024). A systematic investigation into pH regulation in gametogenesis, while also focusing on gamete competence is needed to substantiate this hypothesis.

## Open questions and future directions

In these examples of germline cyst cell death, somatic cells control the cell death and clearance of germ cells in a form of cell death referred to as phagoptosis. However, all phagoptotic deaths may not use the same molecular mechanisms and there is much left to discover. *Drosophil*a NC death is controlled non-autonomously by surrounding epithelial SFCs. These epithelial cells contain lysosomal machinery which has shown to be required for the final steps of NC death, consisting of nuclear membrane breakdown, DNA fragmentation, and acidification (Timmons et al., 2016; Yalonetskaya et al., 2020; Lebo and McCall, 2021). While NC nuclear breakdown has been well-characterized, what remains unclear is how the NC plasma membrane is eliminated. It is possible that prior to acidification, the NC plasma membrane undergoes trogocytosis or cellular “nibbling” (Zhao et al., 2022), by the neighboring FCs. This may provide an efficient mechanism for the much smaller FCs to initiate destruction of the NCs. Recent studies have shown that the SFCs are not the only FCs that can eliminate and clear NCs. Border cells, which normally migrate to the NC/oocyte border in stage 9, can promote the death of NCs when they express hyperactivated Rac (Mishra et al., 2023). These dying NCs contain fragmented nuclei and irregular egg chamber morphology which is dependent on the phagocytic receptor Drpr. Similarly, overexpression of Drpr in main body FCs can drive the death of NCs but this example of phagoptosis drives apoptosis in the NCs (Serizier et al., 2022).

The conservation of non-apoptotic and phagoptotic mechanisms of germ cell elimination is intriguing, yet there are some clear differences and further exploration of the similarities and differences is an exciting avenue for future research. In the *Drosophila* testis, a single cyst cell engulfs the entire cluster of germ cells (Zohar-Fux et al., 2022) whereas in the *Drosophila* ovary, several SFCs surround each individual NC (Timmons et al., 2016; Ghosh et al., 2025). In the fetal mouse ovary, individual NCs are surrounded by pre-granulosa cells, and NCs are acidified asynchronously (Niu and Spradling, 2022), resembling NCs in the *Drosophila* ovary. In the *Drosophila* testis, Rab5-and Rab7-positive phagosomes surround live germ cells prior to acidification (Zohar-Fux et al., 2022), indicating that engulfment is the underlying mechanism of phagoptosis, whereas in the ovary, the process resembles extracellular acidification (Timmons et al., 2016; Mondragon et al., 2019). Nevertheless, there are also acidic vesicles in the SFCs in the *Drosophila* ovary, and transmission electron microscopy has revealed vesicles adjacent to the SFC membrane (Nezis et al., 2000). However, it remains to be determined if these are phagocytic (or trogocytic) vesicles taking up NC material or vesicles releasing their contents into the NCs. Live imaging will be essential to addressing these questions.

The transformation of the cyst cells and stretch follicle cells to a phagocytic fate is of high interest and several recent studies have identified many genes for further investigation. Using high-throughput screening techniques, multiple components of the Toll innate immune signaling pathway were found to affect NC clearance and also egg chamber integrity and survival in mid-to-late oogenesis (Bandyadka et al., 2025). Upstream regulators of Toll such as SPE, PGRP-LC, and PGRP-SD, as well as downstream products of immune activation, antimicrobial peptides, such as Drs, are upregulated in SFCs. Whether components of the Toll pathway play immunogenic rather than morphogenic roles, or achieve both simultaneously, is an exciting line of inquiry which remains to be pursued. Single cell technologies will provide further insight into the gene expression changes in the phagoptotic cells.

It is striking that non-apoptotic and non-cell autonomously controlled germ cell death is found among diverse organisms and in both the testis and ovary. This suggests that there are advantages for utilizing this form of cell death across evolution. Two key advantages of cell non-autonomous over autonomous cell death are, first, the ability to modulate the death rate in response to external cues such as age or nutrient availability, and second, the potential for improved preservation of basic cellular units for recycling. While the *Drosophila* and mouse ovary and testis are valuable models to further investigate these mechanisms given the wealth of genetic tools and tissue accessibility, it will be exciting to see how cell death mechanisms in the germline have evolved across different organisms.
